# Small Saccades and Image Complexity during Free Viewing of Natural Images in Schizophrenia

**DOI:** 10.3389/fpsyt.2013.00037

**Published:** 2013-05-20

**Authors:** Jose Ignacio Egaña, Christ Devia, Rocío Mayol, Javiera Parrini, Gricel Orellana, Aida Ruiz, Pedro E. Maldonado

**Affiliations:** ^1^Laboratorio de Neurosistemas, Programa de Fisiología y Biofísica, Facultad de Medicina, Universidad de ChileSantiago, Chile; ^2^Biomedical Neuroscience Institute, Faculty of Medicine, Universidad de ChileSantiago, Chile; ^3^Departamento de Anestesiología y Reanimación, Hospital Clínico Universidad de ChileSantiago, Chile; ^4^Departamento de Psiquiatría y Salud Mental, Campus Oriente, Facultad de Medicina, Universidad de ChileSantiago, Chile; ^5^Departamento de Psiquiatría y Salud Mental, Campus Norte, Facultad de Medicina, Universidad de ChileSantiago, Chile

**Keywords:** microsaccades, saccades, free viewing, natural images, schizophrenia

## Abstract

In schizophrenia, patients display dysfunctions during the execution of simple visual tasks such as antisaccade or smooth pursuit. In more ecological scenarios, such as free viewing of natural images, patients appear to make fewer and longer visual fixations and display shorter scanpaths. It is not clear whether these measurements reflect alterations in their proficiency to perform basic eye movements, such as saccades and fixations, or are related to high-level mechanisms, such as exploration or attention. We utilized free exploration of natural images of different complexities as a model of an ecological context where normally operative mechanisms of visual control can be accurately measured. We quantified visual exploration as Euclidean distance, scanpaths, saccades, and visual fixation, using the standard SR-Research eye tracker algorithm (SR). We then compared this result with a computation that includes microsaccades (EM). We evaluated eight schizophrenia patients and corresponding healthy controls (HC). Next, we tested whether the decrement in the number of saccades and fixations, as well as their increment in duration reported previously in schizophrenia patients, resulted from the increasing occurrence of undetected microsaccades. We found that when utilizing the standard SR algorithm, patients displayed shorter scanpaths as well as fewer and shorter saccades and fixations. When we employed the EM algorithm, the differences in these parameters between patients and HC were no longer significant. On the other hand, we found that image complexity plays an important role in exploratory behaviors, demonstrating that this factor explains most of differences between eye-movement behaviors in schizophrenia patients. These results help elucidate the mechanisms of visual motor control that are affected in schizophrenia and contribute to the finding of adequate markers for diagnosis and treatment for this condition.

## Introduction

Eye movements are among the most frequent motor acts in human and non-human primates. Our visual perception is constructed from a constant repositioning of the fovea from point-to-point in the visual scene, or by visually following moving objects. Although extremely common, eye movement requires the involvement of extensive areas of the cortex as well as numerous subcortical structures. Since its first description in patients by Diefendorf and Dodge (1908) and later by Holzman et al. (1973), dysfunctional eye movements have been extensively studied in schizophrenia. A large fraction of these studies are devoted to exploring alterations of smooth pursuit eye movements (SPEM), when subjects or patients are instructed to follow a slow moving target (Kumra et al., 2001; Kathmann et al., 2003; O’Driscoll and Callahan, 2008; Nagel et al., 2012). These studies found that schizophrenia patients exhibit deficits in gain of SPEM and impairment in maintaining SPEM velocity, among other deficits. In addition, several other studies have explored eye-movement alteration during voluntary saccadic eye movements. In this paradigm, patients are instructed to perform a voluntary saccade toward a target (prosaccades) or to a direction opposed to the target location (antisaccades). These studies conclude that patients with schizophrenia have problems executing antisaccades, and have significantly greater numbers of antisaccade errors, significantly prolonged latencies of correct responses and self-corrections (Curtis et al., 2001; Kallimani et al., 2009; Mazhari et al., 2011). These alterations arise from the inability of patients to inhibit a natural prosaccadic movement toward the target, which reflects a potential problem in executive control (Hutton et al., 2004; Camchong et al., 2008). A few studies performed in a more ecological setting, where patients freely explored natural or simple visual images, showed that subjects with schizophrenia exhibit reduced exploratory behavior and display fewer and shorter visual fixation or saccades (Beedie et al., 2011).

Underlying many of these studies is the explicit intent to find proper biomarkers for diagnosis and therapeutic evaluation that would complement clinical assessment, and have guided basic studies to understand the neuronal mechanism of this illness. However, given the complexity of the eye-movement behaviors and the intricate neuronal network that participates in eye movements, it seems desirable to determine whether alterations in eye movements in patients diagnosed with schizophrenia arise from low-level mechanisms (Elahipanah et al., 2011). In this case patients would present difficulties in executing motor plans such as saccadic motor movements. Alternatively, these alterations may originate in high-level mechanisms that include planning, motor movements, inhibiting motor action in contextual setting (Lencer et al., 2011), and cognitive dysfunction to engage in aimed movement (Hong et al., 2009). While most studies seem to suggest alterations in high-level mechanisms, reports indicate that basic aspects of eye movements, such as the number and duration of both fixations and saccades are altered, particularly in ecological settings where eye movements are not instructed, but rather freely self initiated by the subjects. The stimulus included in these studies varied from faces (Phillips and David, 1996; Williams et al., 1999; Loughland et al., 2002) to geometrical figures (Kojima et al., 1992; Obayashi et al., 2001) and natural images including fractals and noise (Bestelmeyer et al., 2006; Benson et al., 2007). Studies employing natural images are of interest because this experimental paradigm does not involve a specific task other than to freely explore the images. These studies reported shorter scanpaths displayed by schizophrenia patients, which occurred typically with a reduced number and an increased duration of visual fixations.

Differences reported in these studies, which used different types of images and tasks, suggest that a large component of the differences between schizophrenia patients and healthy controls (HC) relate to high-level cognitive mechanisms. Nonetheless, patients also appear to show a reduced ability to perform saccades and fixations at the rate seen in healthy individuals, pointing to low-level alterations in eye movement. We hypothesized, despite this observation, that the reduced scanpaths, reduced number, and increased mean duration of visual fixations could be related to the detailed methodology utilized to quantify saccades and fixations. This especially concerned a lack of sensitivity in detecting microsaccades. Because of a decreased visual exploration exhibited by patients, an increased occurrence of microsaccades may follow (Collewijn and Kowler, 2008; Martinez-Conde et al., 2009; Rolfs, 2009; McCamy et al., 2012). The main objective of this study is to examine if patients with schizophrenia do exhibit alterations in basic eye movements so that we can better understand whether high or low-level neuronal mechanisms are affected in this group. In this research, we employ free viewing of natural images to elicit the most spontaneous visual behavior in the patients while we include saccades and microsaccades as part of a continuum of eye movements. Rather than aiming at newly developed methodological aspects, we compared the result of applying rigorous eye-event detection algorithms with traditional measures based on basic speed criteria. In order to test our hypothesis, we recorded eye movements during free exploration of natural images in a group of schizophrenia patients and compared visual scanpaths as well as frequency and duration of saccades, microsaccades, and fixations. To contrast more cognitive aspects of visual processing, we presented natural images from seven different categories that differ in image complexity. We found that when small amplitude eye movements, or microsaccades, are taken into account, scanpaths and the number and duration of saccades and visual fixations are largely unaffected in schizophrenia patients. At the same time, we found that these parameters can be strongly affected by the type of image presented to the subjects.

## Materials and Methods

### Participants

This study and written consent documents were approved (N°028-2011) by the Ethics Committee for Research in Humans, of the Faculty of Medicine, Universidad de Chile. We report results from eight patients diagnosed with schizophrenia who met DSM-IV criteria. Patients were outpatients recruited from two mental health centers associated with the University. Detailed medical and psychiatric histories were collected for each person. Experienced psychiatrists using case note reviews and the Structured Clinical Interview for DSM-IV (SCID) (First and Gibbon, 2004) made diagnoses. All schizophrenia patients (SCZ, *n* = 8) were clinically stable and medicated at the time of testing. SCZ patients were excluded if they had a history of head injury or neurological disease(s). The HC group (*n* = 8) included non-clinical individuals recruited from the volunteer panel at the Universidad de Chile and public advertisements. Exclusion criteria comprised responses to semi-structured questions about the history of drug or alcohol abuse, dependence within 6 months before testing, major head trauma, epilepsy, or other neurological dysfunction. All participants, SCZ and HC had normal or corrected-to-normal vision; all volunteers were right-handed and signed an informed consent.

### Experimental procedure

A set of 70 grayscale images was presented on a 21-inch (Viewsonic p815) at a refresh rate of 76 Hz and resolution of 800 × 600 (40° horizontally and 30° vertically with 20 pixels per visual degree). The images were viewed from 57 cm and were presented for 4 s each, in random order. The image set included 10 different images from 7 categories: construction sites, landscapes, fractals, pink noise, white noise, uniformly gray, and black images. The categories as described in this sequence presented decreasing orders of complexity as measured by their spatial frequency power spectra, with construction images having the largest concentration of power at a lower frequency, with an incrementally broadening power spectrum toward the white noise category. Between image presentations, a central fixation point appeared, and the subjects pressed a button to trigger the next image presentation. Participants were instructed to freely explore each image. Infrared eye-movement recording was performed using a head-mounted EyeLink II (SR-Research, ON, Canada) at 500 Hz. Calibrations was performed using a 3 × 3 fixation matrix. Drift correction was applied throughout the recordings. Eye position was sampled binocularly at a spatial resolution of 0.01°.

### Data analysis

**Figure 1:**
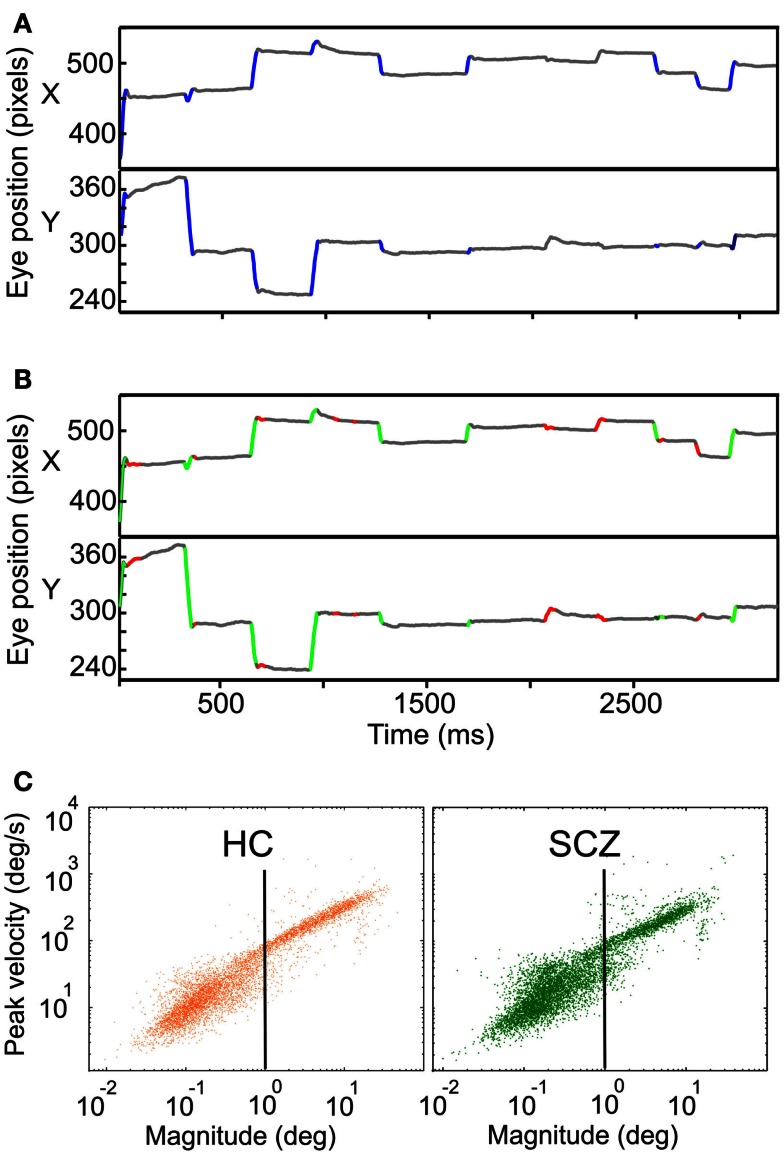
**Examples of eye movement’s recordings showing the SR and EM algorithms implementation**. **(A)** Horizontal and vertical eye traces for a control subject while freely viewing natural images. Traces in blue depict the segments identified as saccades by the SR algorithm. **(B)** Same vertical and horizontal eye traces shown in **(A)**. Here, traces in green and red depict the segments identified by the EM algorithm as saccades and microsaccades, respectively. **(C)** Main sequence of saccades and microsaccades for HC (in orange) and SCZ (in green) using the EM algorithm (*n* = 6867 and 6431, respectively). The vertical black line depicts the 1° boundary between saccades and microsaccades.

Before employing any eye movements identification method on the eye-tracking data, we corrected blinks duration based on the EyeLinkII manufacturer’s user manual. On the corrected data, we used two different methods for detecting and measuring saccades and fixations. A first analysis used the information contained in the SR-Research output archives (hereinafter called SR algorithm), by which we identified saccades and fixations periods. The SR algorithm established saccades based on a velocity threshold of 30°/s and an acceleration threshold of 8000°/s^2^. With these parameters mostly saccades bigger than 0.5° were identified. In a second analysis, we identified saccadic movements based on an adaptation of an objective method proposed by Engbert (2006), called hereafter the EM algorithm. Briefly, based on the instantaneous position of the eye, velocity was derived and a velocity threshold was set. The velocity threshold was four times the standard deviation of the previously computed velocity data (λ = 4). This threshold was calculated for horizontal and vertical eye data independently. Then, in a velocity state space (*v_x_*, *v_y_*), these horizontal and vertical velocity threshold set a region where any velocity point lying outside was identified as a possible microsaccade/saccade. Afterward, the data was further classified as saccades, or microsaccades based on the eye movement amplitude where microsaccades displayed less than 1° of amplitude. The EM algorithm was originally proposed for a fixation task; thus the EM algorithm was applied only during fixations periods already detected by the SR algorithm. For the two methods of saccade/microsaccade detection used here, we verified they were binocular movements and set a minimum duration of 6 ms for saccades and 20 ms for fixations (Martinez-Conde et al., 2004). Figure 1 shows an example of the SR (A) and EM algorithm (B) implementation for the same data set. In the last method, fixations were defined as time events that were neither saccades/microsaccades nor blinks. We also computed the main sequence (Otero-Millan et al., 2008) for all saccades/microsaccades for controls and patients (Figure 1C).

In order to quantify exploration, we used two different computations: Euclidean distance and scanpath. Euclidean distance was defined as the minimal distance, in pixels, between two successive fixations (straight line). On the other hand, scanpath was measured as the actual pathway followed by each eye between two successive fixations, meaning that overshoots and eye corrections performed during a saccade were considered in this measure. We use mean value between both eyes to quantify visual exploration (Euclidean distance and scanpath). For each subject we add the total distance among images of the same category.

### Statistics

To compare variables of interest, central tendency measures were computed for each subject’s data. Then, between group and within group *t*-tests were performed over the two samples. Distribution differences were assessed by a two-sample Kolmogorov–Smirnov test.

## Results

### Participants

We performed eye movement measurement in 12 patients (SCZ) and 8 HC subjects in this report. Four patients were excluded from the study because the eye data recordings were excessively noisy due to frequent eye blinks. Table 1 shows a summary of the demographic and medical features of the subjects. Qualitative assessment of IQ was performed. Either their psychiatrists, in the case of patients, or a trained evaluator, in case of controls, performed this evaluation. All subjects, patients, and controls, were cataloged either as average/normal or high average. Socioeconomic situation was calculated using the deciles corresponding to the area of the city where each participant lives. Median and interquartile ranges for that decile data were calculated. Medical information was added for the SCZ group. One patient’s data was not retrieved because we could not access her medical file. The type and amount of medication at the time of the recordings were also registered. We calculated chlorpromazine equivalent doses (daily) based on Kroken et al. (2009). All medication was administered orally (PO).

**Table 1:** **Demographic and medical features of SCZ and HC groups**.

	Schizophrenia patients	Healthy controls
Subjects (*n*)	8	8
Age in years (mean ± SD)	33.9 ± 11.4	32.4 ± 13.9
Gender (M/F)	6/2	5/3
SE median (IQR)	9 (4.5)	5.5 (4.5)
Months since diagnosis	104 ± 86	NC
Paranoid schizophrenia^*^	7/8	NC
FGA	2/8	NC
SGA	4/8	NC
FGA + SGA	1/8	NC
AED (daily mg of CPZ)^**^	296 ± 87	NC

SE, socioeconomic decile; IQR, interquartile range; FGA, first generation antipsychotics; SGA, second generation antipsychotics; AED, antipsychotic equivalent dose. ^*^One patient data could not be retrieved. ^**^Based on Kroken et al., 2009 (see Materials and Methods).

### Main sequence

The velocity of a saccade linearly depends on its amplitude, a relationship known as the main sequence. We plotted the peak velocity versus the magnitude of every microsaccade/saccade as computed with the EM algorithm, for all HC and SCZ patients. This relationship is presented in Figure 1 where we plotted the main sequence for HC and SCZ patients (*n* = 6867 and 6431, respectively). These plots demonstrate a continuum in microsaccades and saccades parameters and suggest that such basic features of eye movements in SCZ patients are remarkably similar to those of HC.

### Euclidean distance and scanpaths

**Figure 2:**
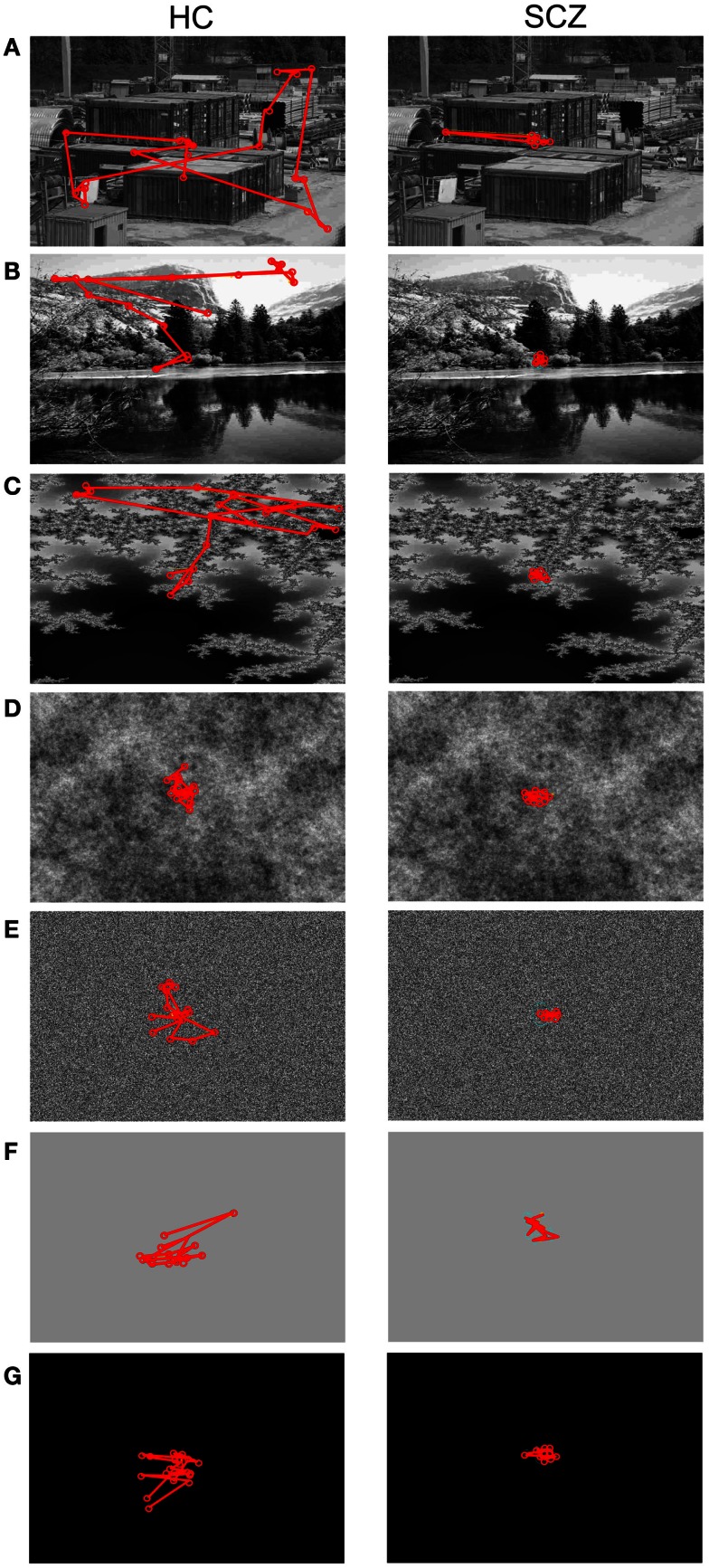
**Visual exploration of images with different complexity**. Here are examples for a healthy control (HC, left column) and a patient affected with schizophrenia (SCZ, right column). The red lines and circles depict saccades and fixations, respectively. The example images are sorted in decreasing complexity measured as variances of their spatial frequency power spectrum. **(A)** Construction, **(B)** landscape, **(C)** fractals, **(D)** pink noise, **(E)** white noise, **(F)** gray, and **(G)** blank images. Both subjects reduce the area covered by visual exploration as image complexity falls, but SCZ subjects typically exhibit more reduced areas than HC participants.

The large majority of previous studies reported that SCZ patients show heterogeneous visual exploration patterns for different types of images. An example of each image category is shown in Figure 2. It is apparent in this instance that the SCZ patients covered a smaller visual area during visual exploration compared to healthy volunteers.

**Figure 3:**
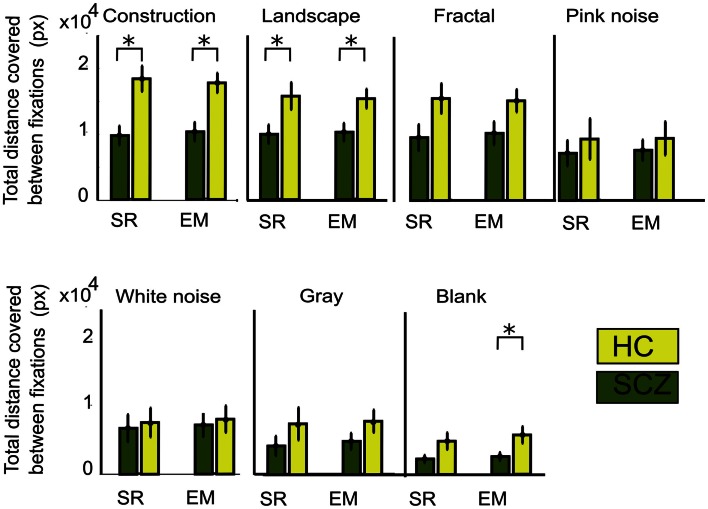
**Total Euclidean distance for different image category**. The measure for visual exploration adds the minimal distances between fixation locations (see Materials and Methods) measured with the SR and EM algorithms. The total average distance for each subject is shown for each image category. In both groups of subjects, the amount of distance decreases with image complexity, independent of the algorithm used. SCZ patients showed significantly reduced visual exploration. The EM algorithm still shows significant differences for constructions, landscapes, and blank images. Error bars indicate SEM (*p < 0.05 or less, for two-sample *t*-test).

As a measure of image exploration, we computed total distance covered for SCZ and HC subjects for each image category. Euclidean distance is the length of a straight line between successive fixations, while scanpath is the actual pathway covered by eyes considering overshoots and eye corrections. In Figure 3, we show the mean total Euclidean distance covered by visual exploration in the SCZ patients (green) versus HC groups (yellow). As reported in other studies, visual exploration depends on the type of images explored (Jansen et al., 2009; Berger et al., 2012). The largest distances were found for the constructions and landscapes while the smallest distances were found for the gray and blank images. We also show that regardless of image category, Euclidean distances for SCZ patients always had lower magnitudes than HC patients. However, significant differences were found only for constructions (*p* < 0.01) and landscapes (*p* < 0.03) images using the SR method. These differences were maintained when we used the EM algorithm to compute eye movements and microsaccades. Significant differences were again found for constructions (*p* < 0.01) and landscapes (*p* < 0.03), but also for blank images (*p* < 0.03). These results imply that the expected level of exploration could differ significantly for images with different complexity. For instance, SCZ patients explore as much in construction images as HC patients do in pink noise images (*p* = 0.91). They also suggest that cognitive components related to the nature of the images are clearly important in modulating visual exploratory behaviors.

**Figure 4:**
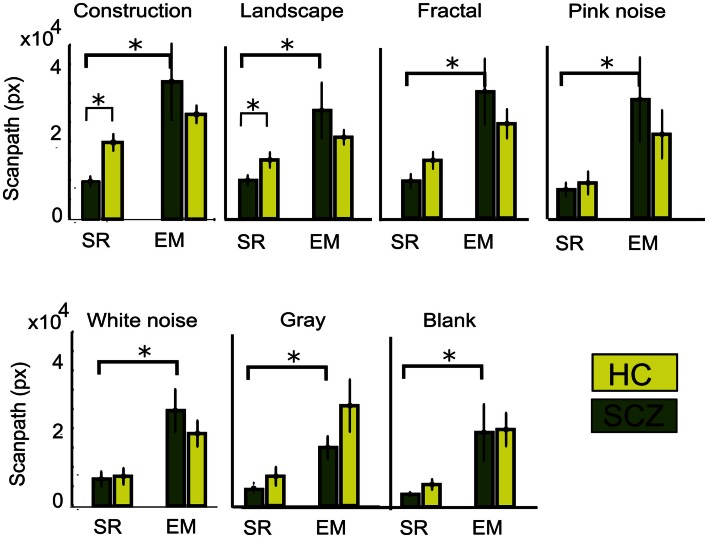
**Total scanpath for different image category**. This parameter measures the actual distance covered by the eye’s movements. When the EM algorithm is employed, the scanpath increases significantly for every image category, but there was no significant difference between SCZ and HC groups. Error bars indicate SEM (*p < 0.05 or less, for two-sample *t*-test).

The distance measured between saccades does not entirely account for the total distance covered by the eye movements. Figure 2 gives the impression that because patients explored considerably less than controls, it follows that the actual distance followed by the eyes must be longer in controls than in patients. However, inspection of Figure 3 is misleading in this regard because microsaccades occur often during what appears to be a long visual fixation. In addition, while Euclidean distance measures the minimal distance covered by the eyes between fixation points, eye movements do not always follow a direct route. For instance, frequent overshoots can substantially add length to the distance between fixations and constitute markers for neurological alteration (Bahill et al., 1975). Therefore, we computed the scanpaths for all subjects by measuring the actual distance covered by the eyes during visual exploration (Figure 4). We found that, as occurred with Euclidean distances, scanpaths performed on all types of images by the SCZ groups were smaller than the HC group when computed with the SR algorithm. These differences were significant for the construction images (*p* < 0.01) and landscapes (*p* < 0.04). But when the EM algorithm was used, we found that while scanpaths increased for both groups, there were no longer differences between both groups. Furthermore, when microsaccades were taken into account (EM algorithm), the scanpaths of the SCZ patients increased significantly for all image categories (*p* < 0.02, 0.02, 0.01, 0.05, 0.01, 0.01, and 0.04; for constructions, landscapes, fractals, pink noise, with noise, gray, and blank images, respectively). In contrast to Euclidean distances, which were image dependent, scanpaths distances were similar (no significant differences) for all image categories. These results demonstrate that SCZ patients exhibit the same regularity of eye movement than HC subjects, but differences in visual exploration reported in previous studies appear to result from the exclusion of small eye movements. Here we show that scanpaths are similar for different image categories while Euclidean distances appear to depend on the cognitive nature of the explored image.

### Saccades frequency and duration

**Figure 5:**
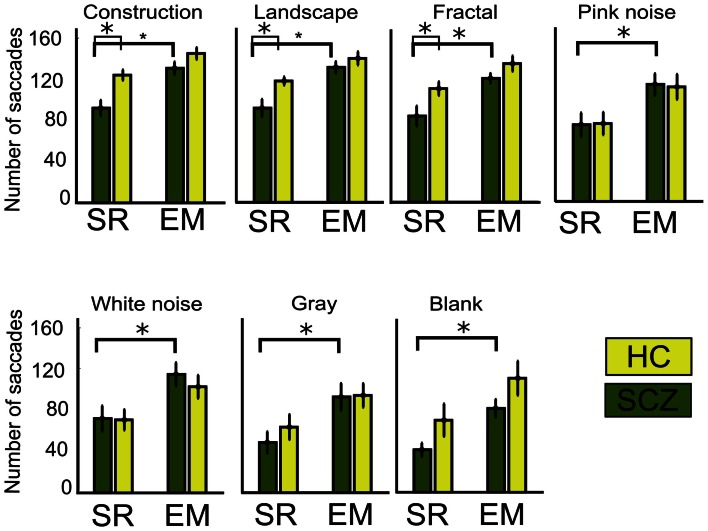
**Number of saccades for different image category**. Graph shows the average, for each group, of the total number of saccades performed while viewing the ten images of each category. When the SR algorithm is employed, significant differences are found for constructions, landscapes, and fractals images only. Employing the EM algorithm significantly increases saccades for all image categories, but the number for saccades is similar between SCZ and HC groups. Error bars SEM (*p < 0.05 or less, for two-sample *t*-test).

In this study, we conjectured that decreased visual exploration leads to apparent shorter scanpaths because of undetected microsaccades. In order to examine this question, we computed the number of saccades as measured by the SR and EM algorithms (see Materials and Methods for details). When employing the SR algorithm, we observed that SCZ patients effectively displayed a reduced number of fixations when compared to HC. These differences were significant for constructions, landscapes, and fractals (*p* < 0.00, 0.01, and 0.04, respectively), but not for pink and white noise or for gray and blank images. Figure 5 shows the average number of saccades performed for each image category for the SCZ groups. When we employed the EM algorithm, the number of saccades for the SCZ group increased significantly for all image categories (*p* < 0.01, 0.01, 0.01, 0.02, 0.02, 0.02, and 0.01; for constructions, landscapes, fractals, pink noise, with noise, gray, and blank images, respectively). Moreover, the number of saccades computed with this algorithm was similar to those for HC participants for all image categories. This result clearly indicated that both groups of subjects perform a large number of small saccades that are undetected by the regular SR algorithm and that differences between groups tend to fade away when we employed the EM algorithm. Additionally, we compared the median duration for the saccades performed by both groups of participants (data not shown). We found that the duration of saccades computed with the SR algorithm, for SCZ and HC groups, was similar for all types of images. When we employed the EM algorithm, we verified a consequent reduction of the median for the saccade distribution for the SCZ group, albeit only significant for construction images (*p* < 0.05). These results suggest that the median duration of saccadic eye movements does not appear to be altered in SCZ patients and that the inclusion of undetected microsaccades maintains this finding. These data also suggest that the complexity of the image does not modulate the median saccadic duration, in contrast to what occurs with visual exploration.

### Fixations frequency and duration

**Figure 6:**
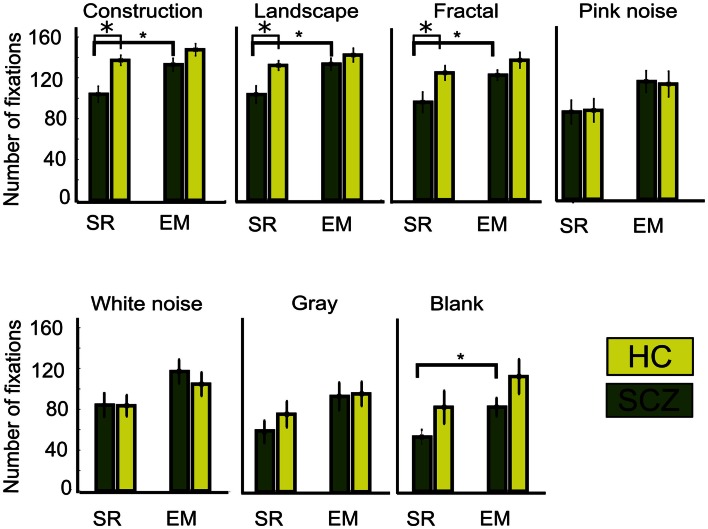
**Number of fixations for different image category**. This graph shows the average, for each group, of the total number of fixations performed while viewing the ten images of each category. As occurred with the saccades, when the SR algorithm is employed, significant differences are found for constructions, landscapes, and fractals images only. Employing the EM algorithm significantly increases the number of fixations for the same image categories and also for the blank. With EM, the number for fixations is similar between SCZ and HC groups. Error bars SEM (*p < 0.05 or less, for two-sample *t*-test).

A large number of studies have reported that SCZ patients display a reduction on the number of visual fixation together with an increment in their duration (Beedie et al., 2011). In this study, we compared the SR and EM algorithms in their quantification of these parameters. We observed that when measuring the number of fixations with the SR algorithm, indeed, SCZ patients display a reduced number of fixations when compared to HC. Figure 6 shows the average number of visual fixations performed for each image category for the SCZ and HC groups. Differences were significant for constructions, landscapes, and fractals (*p* < 0.01, 0.01, and 0.03, respectively) but not for pink and white noise, as well as for gray and blank images. Figure 6 plots show that the number of fixations performed by SCZ participant was typically smaller than that performed by HC individuals in the image categories with higher complexity. For blank images, the total number of fixations was about half the number of other image categories with higher complexity, such as constructions, landscapes, and fractals. In contrast, when the number of fixations is quantified using the EM algorithm, the average number of fixations in each image category increased. This result can be expected from the EM algorithm because the detection of microsaccades would break fixations in two or more segments, increasing their number and reducing their average duration. As can be seen in Figure 6, this was the case for constructions, landscapes, fractals, and blank images (*p* < 0.01, 0.01, 0.03, and 0.02, respectively). Moreover, for all image categories, the differences observed between SCZ and HC groups were no longer significant, but image complexity or cognitive content still impact the number of visual fixations.

**Figure 7:**
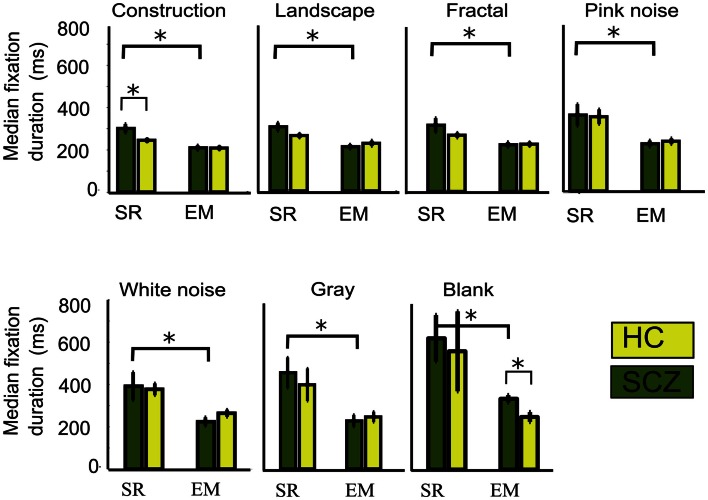
**Median fixations duration for different image category**. We plotted the median fixation duration for each experimental group while viewing every image category. Here, only significant differences for the SCZ and HC groups are found for the constructions images when the SR algorithm is employed. When SR is compared with EM, significant differences are found for all image categories in SCZ, but only presentation of blank images elicited significant differences between SCZ and HC groups under EM algorithm. Error bars indicate SEM (*p < 0.05 or less, for two-sample *t*-test).

When we examined the duration for fixations performed by the SCZ versus the HC groups (Figure 7), we found that only in the construction image category (largest image complexity) SCZ patients displayed significantly longer fixation medians for the SR algorithm (*p* < 0.01). On the other hand, as the number of fixations increased with the EM algorithm, a reduction of the fixations duration can consequently be expected. Figure 7 shows the median duration for fixations, for all image categories for both the SCZ and HC groups. We found that by using the EM algorithm, the median duration of fixations in each image category for the SCZ group decreased for all image categories (*p* < 0.01, 0.01, 0.02, 0.02, 0.03, 0.01, and 0.02; for constructions, landscapes, fractals, pink noise, with noise, gray, and blank images, respectively). Interestingly, fixations duration for SCZ and HC groups were similar for all images, except for the blank category. Here, SCZ subjects showed significantly longer durations than HC participants (*p* < 0.01). These results demonstrate that when the EM algorithm is being used, the differences observed in the number and duration of fixation of SCZ patients on images with high complexity (as constructions) are no longer significant, and that median duration for SCZ and HC converge to similar values. These also suggest that when precise measurements of eye movements are employed, which include microsaccades, the largest impact of observable differences in saccades and fixation values relates to the complexity of the images, rather than intrinsic eye movements mechanisms.

### Fixations duration distribution and image categories

**Figure 8:**
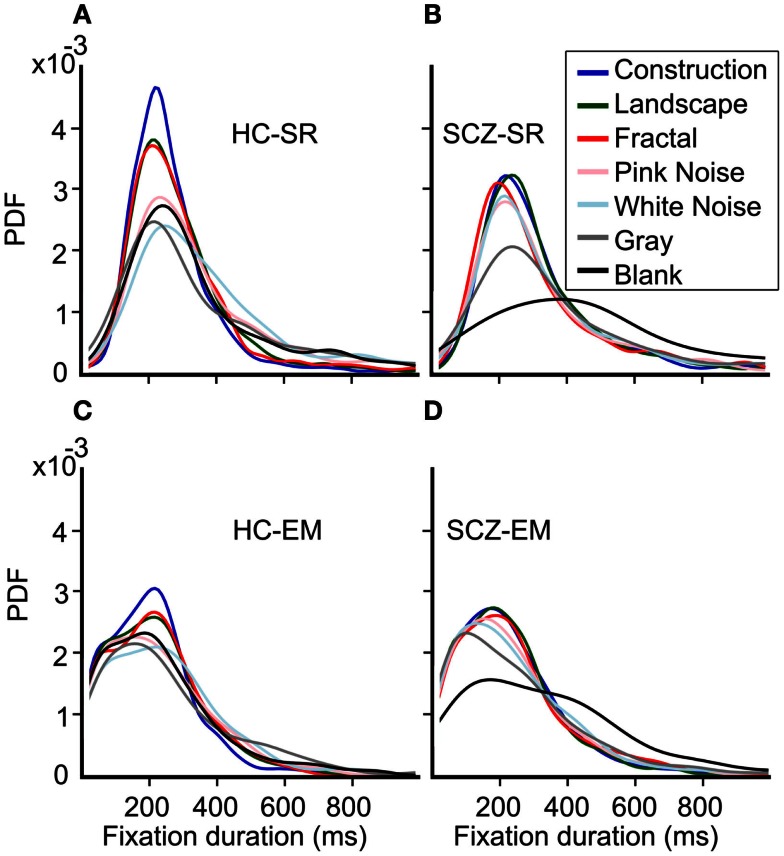
**Probability density function (PDF) for fixation durations for each image category**. **(A–D)** These plots are a smoothed representation of the binned histograms. HC subjects show a narrower distribution for fixation durations using the SR algorithm for image categories with the largest complexity. The distribution for construction was significantly different from all others; landscapes and fractals were similar but different to the other categories which were similar to each other (Kolmogorov–Smirnov, *p* = 0.05). When we employed the EM algorithm, we found that the mode of their distribution changes slightly, with a largest proportion of the fixation duration seen for durations below 200 ms. However, statistical differences between these curves remain identical to those observed for the SR algorithm.

**Figure 9:**
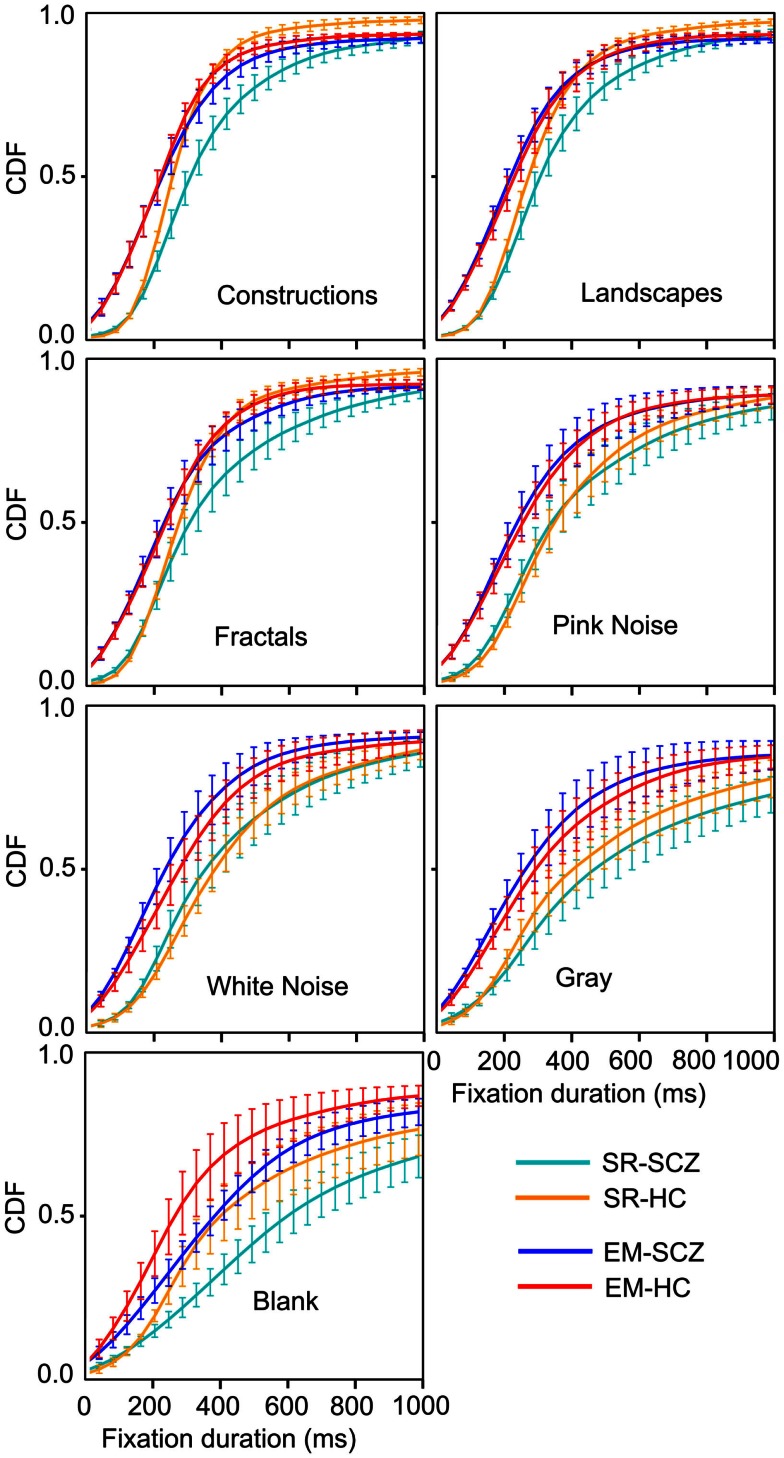
**Cumulative density function (CDF) for fixation durations for each image category**. These graphs show the shift in fraction fixation with short and large duration. The brackets depict the 95% confidence interval for each fixations duration bin. Employing the EM algorithm resulted in a left-shift of the distribution, both for the HC and SCZ subjects. Moreover, differences between these groups that were observed for images with high complexity were no longer significant when the EM algorithm was employed. Nevertheless, blank images still show significant differences in the distribution of fixation duration between SCZ and HC groups.

Because several previous studies have reported significant differences in fixations duration when comparing SCZ and HC groups, we further compared the distributions for fixations duration for all images and groups. Figure 8 shows the probability density function (PDF) for fixation durations obtained for each image category as computed by both the SR and EM algorithms. These plots are a kernel-smoothed representation of the binned histograms. We found that in HC subjects, the narrower distribution for fixation duration using the SR algorithm was found for image categories with the largest complexity (Figure 8A, constructions, landscapes, fractals) while others’ image categories evoked fixations distributions with wider ranges. We found that the distribution for construction was significantly different from all others and that landscapes and fractals were similar to each other but different from the other categories (Kolmogorov–Smirnov, *p* ≤ 0.05). When we computed the PDF plots for the SCZ patients, we found that they differ significantly from the HC individuals (Figure 8C) (Kolmogorov–Smirnov, *p* ≤ 0.05); the mode of the distribution does not change substantially for most image categories but the blank; and the distribution of SCZ’s data is wider. In this case, we found that their distribution for constructions, landscapes, fractals, pink noise, and white noise was similar but statistically different from gray and black images, which were also significantly different from each other (Kolmogorov–Smirnov, *p* ≤ 0.05). This was confirmed when we computed the cumulative density functions (CDF, Figure 9) that showed that the differences in the fixation duration distributions seen in Figures 8A,C were related to fixation duration that exceeded 200 ms. Notably, the distribution of fixations duration for blank images is the widest with a mode about twice as large as the mode for fixations duration in other image categories. When we employed the EM algorithm, we found that the mode of their distribution changes slightly, with the largest proportion of the fixation duration seen for durations below 200 ms (Figures 8B,D). This result is mostly a consequence of finding microsaccades in long fixations. Despite the change in the PDF’s shapes while using EM algorithm, the statistical differences between these curves remain identical to those observed when the SR algorithm was employed. As with the HC subject, the PDF plots computed with the EM algorithm show an increment in the fraction of fixations duration with lower values. Also, the similarity of plots between the images remains unchanged, with the gray and black images significantly different from all other groups and between each other.

The CDF functions shown in Figure 9 illustrate more clearly the differences between the SCZ and HC groups. For all image categories, curves computed with the SR algorithm (cyan and yellow curves) show a similar fraction of low fixation durations but then show that the SCZ group displays longer fixation durations lasting more than 200 ms. When the EM algorithm is utilized (blue and red), all plots show a leftward shift, indicating that there was a larger fraction of shorter fixations duration. The differences between the SCZ and HC groups were not significant any longer, as it is demonstrated by error bars that show the 95% confidence interval of the data at that time. Again, a notable exception occurs during the presentation of blank images where the SCZ group significantly displayed longer fixation than the HC group. Most of the differences between SCZ and HC with the SR algorithm were found in image categories with large complexity (i.e., construction, landscapes, and fractals). However, with the EM algorithm, this phenomenon is no longer observed. It is apparent, though, that the SCZ groups, in the absence of an image, effectively perform longer visual fixations regardless of the algorithm employed.

## Discussion

### Saccadic movements: Microsaccade-saccade continuum

In our study, we employed two algorithms to compare eye movement behavior between schizophrenia patients and HC. For this purpose, we compared the regular SR algorithm with another that detects large and small eye movements in order to assess saccade-microsaccades continuum. Both HC and schizophrenia patients make a large number of microsaccades, with patients possibly performing a larger proportion of microsaccades from the total eye movements. Microsaccades are small saccades that are produced during attempted fixation (Martinez-Conde, 2006). They occur with high frequency in a visual task that requires keeping fixations in a small region of the screen for long periods. On the contrary, microsaccades occur infrequently on the free-viewing paradigm (Maldonado et al., 2008; Otero-Millan et al., 2008). In addition, the main sequence plots built with the EM algorithm resulted in similar shapes and in a continuous distribution of microsaccades and saccades. These plots demonstrate that the saccades/microsaccades parameters for both, patients and controls, are similar and argue in favor of the saccade/microsaccade continuum.

The idea of a common physiological generator of saccades and microsaccades, which are what we have all grouped into saccadic movements, is not new (Zuber et al., 1965). At times, microsaccades were overruled as laboratory artifacts. However, in the latter years, new evidence has restored its possible functional role in a wide variety of perceptual tasks with eyes fixating (van Dam and van Ee, 2006; Laubrock et al., 2008; Troncoso et al., 2008). Otero-Millan et al. (2008) have shown that microsaccades are also present in free-viewing tasks. They showed that the parameters of microsaccades dynamics change with stimulus and tasks. It has been shown that eye movement properties, such as saccade and fixation duration, change with second-order stimulus properties (Jansen et al., 2009). In their study, Otero-Millan and colleagues evidenced that both movements followed the same main sequence, even under different task conditions. Altogether, their results suggest the same functional role for small saccades (microsaccades) and saccades, arguing in favor of a saccadic movement continuum in free viewing (Martinez-Conde et al., 2013). Indeed, when studying the parameters of saccades and microsaccades during visual exploration, many common features are shown; they are binocular movements of similar amplitude and direction, they follow a common main sequence, intersaccadic intervals are comparable to inter-microsaccadic intervals in different tasks, movements can be suppress voluntarily, and microsaccades/saccades have a role in attention shifts (Martinez-Conde et al., 2009). Physiological evidence shows that in primate’s superior colliculus (SC), downstream neurons were active during saccades and microsaccades generation (Van Gisbergen et al., 1981; Hafed et al., 2009; Hafed and Krauzlis, 2012). Specifically, Hafed et al. (2009) evidenced that neurons in the foveal part of the SC had selectivity for amplitude and direction of microsaccades. They suggested a causal role of SC in microsaccades generation, which has been previously showed for saccade generation (McPeek and Keller, 2004). The properties of the behavioral continuum between saccades and small saccades are also a shared neuronal activity of SC related neurons but with some neurons showing changes in activation in saccade and microsaccade movements (Hafed and Krauzlis, 2012). Because of the frequency and regularity of saccades and microsaccades, Schroeder et al. (2010) suggested that this rhythmic motor activity could be instrumental in active sensing. Some of its controversial role during viewing was mainly a product of misleading identification of these movements, explaining the broad range of parameters studied as saccade number or fixation duration, among others (Martinez-Conde et al., 2004). A recent report arguably demonstrates that microsaccades and saccades are, in fact, a continuum of the same physiological mechanisms, mainly because they share a common generator (Martinez-Conde et al., 2013).

Overall, the inclusion of microsaccades is important in this study because we are evaluating whether basic eye movements are altered in schizophrenia. By detecting and grouping saccades and microsaccades, we can examine all movements that result from a common generator mechanism. Thus, considering all saccadic movements from schizophrenia patients and HC seems necessary to assess their true behavioral differences. The microsaccade identification method used here (Engbert and Kliegl, 2003; Engbert, 2006) is an objective identification algorithm that relies on statistical measures of velocity to determine saccadic/microsaccadic movements, but still the boundary to distinguish between both movements is somehow arbitrary.

### Visual exploration and scanpaths of natural images in schizophrenia

In the present study, we report that schizophrenia patients exhibit less image exploration when compared with HC. These findings reproduce what has been reported previously under similar experimental settings (Bestelmeyer et al., 2006), suggesting a robust and conserved abnormality. There was something interesting in our results: when scanpath and not Euclidean distance between fixations is considered, there are no significant differences between SCZ and HC subjects. These data show that processes that regulate the numbert of movements seems to be unaffected by this illness, and that is the control of exploratory behavior what it is severely altered in SCZ, thus suggesting more cognitive or high-level deficits.

Among saccades and microsaccades in schizophrenia patients, it has also been shown that when using traditional approaches to assess saccades numbers (SR method), patients make fewer saccades compared to HC (Gooding and Basso, 2008). Microsaccades are small eye movements (<1°) produced during fixation periods. There is evidence that supports the idea that microsaccades and saccades have equivalent functional roles (Martinez-Conde et al., 2004; Otero-Millan et al., 2008). To our knowledge this is the first report that considers microsaccades in a group of patients affected with schizophrenia. Microsaccades/saccades assessment as a unified phenomenon allowed us to observe how the number of saccades was similar between SCZ and HC participants for all image categories. This finding further supports the idea that low-level motor controls are not affected in schizophrenia.

### Number and duration of fixations

Previous data have shown that SCZ patients display a reduction on the number of visual fixation and an increment in their duration (Matsushima et al., 1998; Loughland et al., 2002; Mikami et al., 2003). In this study, we found similar results when we used SR algorithm, but these results changed when we considered the EM algorithm. The latter analysis results in two observable effects in fixation occurrence. First, the number of fixations increase for both groups. This is highly expected since the number of saccades is higher for both groups as we also detected the small saccades. Second, the differences between groups for complex images disappear; the opposite is found for blank stimuli. A similar effect is observed for fixations’ median duration. One interesting observation is the fact that when EM is applied to fixations, control subjects maintain differences between images with decreasing cognitive content (Jansen et al., 2009). In an opposite way, SCZ patients do not alter their “indifference” to explore images, exhibiting similar fixation distribution for all images (except blank). Fixation behaviors in the schizophrenia population point in the same direction than saccades and scanpath analysis: differences between SCZ and HC are mainly due to high-level mechanisms that control, among other things, differential eye movements’ behavior for different images complexity.

### Low versus high-level visual control in schizophrenia

Eye movements as motor acts are the result of the integration of numerous neuronal processes related to various regions in the central nervous system. Related structures include subcortical areas such as the nucleus reticularis tegmenti pontis (Büttner-Ennever and Horn, 1997), SC (Gandhi and Katnani, 2011; Shen et al., 2011), putamen (Burke and Barnes, 2006), thalamus (Wurtz et al., 2011), and cerebellum (Voogd et al., 2010). All of these structures act in a coordinated manner with large cortical areas (Pierrot-Deseilligny et al., 2004) to control eye movements. Two major modulators influence all this neuroanatomical interaction: pre-attentional phenomena determined mainly by image features such as contrast, luminosity, and spatial frequency (Itti and Koch, 2001) and attention-driven activity (Fries et al., 2001), which, in turn, can be affected by other cognitive processes such as memory, emotions, and semantic processing (Noudoost et al., 2010). The sensory-driven control of eye movements is related to a network that includes the striatum, thalamus, SC, and primary visual cortex. Cognitive control, especially volitional saccades, is related to cortical areas that are also important in “bottom-up” processes such as the frontal and supplemental eye-field (FEF and SEF) but seems to be more active in “top-down” paradigms (McDowell et al., 2008). Although low versus high-level should not be confused with bottom-up and top-down, both definitions share the idea that eye movements are under the influence of two mechanisms that differ largely in complexity and are activated in different manners depending on the task to which the system is exposed (Einhäuser et al., 2008a,b). As stated previously, most of the eye movement research in schizophrenia has used pro/anti saccades and smooth pursuit paradigms (Levy et al., 1993; O’Driscoll and Callahan, 2008). These latter approaches, although useful, present at least two caveats in that they do not assess gaze control mechanisms during natural stimuli and free viewing. Stimulus saliency exerts an important influence on fixation localization (Peters et al., 2005). Thus, using unnatural stimuli such as those used in traditional studies does not permit the exploration of the influence of the bottom-up mechanism in schizophrenia dysfunctions. In the same way, free exploration allows the exploration of a more broad effect of cognition in saccadic control since other aspects of cognition are more prompted to arise in a no-goal task (Berger et al., 2012). In addition, exploiting the frequently occurring eye movements has the advantage that the number of observations obtained from one subject is much larger than the traditional paradigms. This enabled the inclusion of a reduced number of subjects to obtain statistically significant observations as occurred in this study. The second issue with traditional paradigms is that new aspects of eye movements’ motor control, such as the study of microsaccades and its relevance, are restricted (Pastukhov et al., 2012). Both difficulties limit conclusions obtained regarding whether there is a low or high-level (or both) dysfunction in schizophrenia. In this work, we combined a free exploration of natural images with an analysis that takes into account small involuntary movements. We showed that patients and HC exhibited similar motor behavior in terms of saccades and fixations when we considered small movements. We also found that it was not the amount of movement but the total exploration that seems to be altered in SCZ. Finally, this study demonstrated that image content does not modulate exploration in the group affected with schizophrenia, despite normal eye motor behavior, and that high-level aspects of eye movements’ control are primarily affected by this illness.

### Image complexity and cognitive content

There is substantial evidence that some images’ features are processed at very early levels of perception (Horwitz and Newsome, 2001; Krauzlis et al., 2004). There is also experimental data that show that image properties can modulate higher-level computations that will, in turn, affect previous mentioned early levels (Naber et al., 2012). In this work, we presented patients seven different categories of images with decreasing levels of complexity determined by their spatial frequency spectrum (data not shown) in order to assess how these properties influenced patients’ eye movements. We found that, although SCZ patients displayed similar scanpaths and performed as many fixations as HC participants, their exploratory behavior appears not to be affected by image properties. In this regard, appears important to learn how faces, as a special type of natural images, impact the parameters examined in our study. Since early works from Yarbus (Tatler et al., 2010), faces have stood out as a special recognition paradigm for which human visual motor and recognition systems seem to be highly specialized (Pascalis and Kelly, 2009). Although there are many studies using face stimuli in patients affected with schizophrenia (see Beedie et al., 2011), their physical properties of spatial frequency components and visual saliency are often at odds with the pattern of visual exploration (Berger et al., 2012).

### Significance of eye movements as markers for schizophrenia

Effectiveness of schizophrenia treatments depends on disease timing, early treatment leading to a better outcome (Lieberman et al., 2001; Wykes et al., 2009). The search for a preemptive treatment of schizophrenia risk patients is on progress, with already encouraging results in animal models (Lee et al., 2012). The possibility that cognitive training before clinical schizophrenia onset might reduce or even prevent cognitive dysfunction comes with the need of schizophrenia detection before clinical symptoms’ onset. Eye movements may turn out to be part of the mild disturbances on adolescents before a full clinical criterion is met. The evidence of a possible high-level disorder in the patients’ cognitive organization, with a probable preservation of low-level control of eye movements, further argues in favor of a cognitive treatment for preclinical schizophrenia patients. Nonetheless, it is important to note that patients are medicated, and while the medication differs among patients, the potential contribution of specific drugs to alteration of eye movements at a high-level control is yet to be determined.

Overall, the results presented in this study strongly indicate that patients with schizophrenia do not exhibit deficits in their abilities to perform basic eye movements. When small amplitude eye movements are taken into account, we showed that scanpaths, the number and duration of saccades and visual fixations, were largely unaffected. At the same time, we observed that these parameters could be strongly affected by the type of image presented to the subjects, both for healthy and subjects affected with schizophrenia. Therefore, there is still substantial potential to continue to use these behavioral markers to differentiate between patients affected by schizophrenia and other mental illnesses. Eye movements in natural vision are unconsciously self-initiated and occur frequently, enabling efficient statistical measurements. We conclude that a better knowledge of the cognitive aspects involved in the visual exploration of the different categories of natural images, combined with eye movement studies, can prove a proficient combination in the search for biomarkers in schizophrenia and other mental illness.

## Conflict of Interest Statement

The authors declare that the research was conducted in the absence of any commercial or financial relationships that could be construed as a potential conflict of interest.
